# Crystal structure of 2-chloro-3-(di­meth­oxy­meth­yl)-6-meth­oxy­quinoline

**DOI:** 10.1107/S205698901500804X

**Published:** 2015-04-30

**Authors:** Nanjappa Chandrika, Tholappanavara H. Suresha Kumara, Jerry P. Jasinski, Sean P. Millikan, Hemmige S. Yathirajan, Christopher Glidewell

**Affiliations:** a PG Department of Chemistry, Jain University, 52 Bellary Road, Hebbal, Bangalore 560 024, India; b University B.D.T. College of Engineering (a Constituent College of VTU, Belgaum), Davanagere 577 004, India; c Department of Chemistry, Keene State College, 229 Main Street, Keene, NH 03435-2001, USA; d Department of Studies in Chemistry, University of Mysore, Manasagangotri, Mysore 570 006, India; e School of Chemistry, University of St Andrews, St Andrews, Fife KY16 9ST, Scotland

**Keywords:** crystal structure, quinolone, pseudosymmetry, twinning, π–π stacking inter­actions

## Abstract

The title compound, C_13_H_14_ClNO_3_, crystallizes with *Z*′ = 2 in the space group *Pca*2_1_, but a search for possible additional crystallographic symmetry found none. However, the crystal structure exhibits pseudosymmetry as the two independent mol­ecules are related by an approximate but non-crystallographic inversion located close to (0.38, 0.26, 1/2) in the selected asymmetric unit, and the structure exhibits partial inversion twinning. The approximate inversion relationship between the two mol­ecules in the selected asymmetric unit is clearly shown by comparison of the relevant torsion angle in the two mol­ecules; the corresponding torsion angles have similar, although not identical magnitudes but with opposite signs. The mean planes of the quinoline rings in the two independent mol­ecules are almost parallel, with a dihedral angle of only 0.16 (3)° between them, and the mutual orientation of these rings permits significant π–π stacking inter­actions between them [centroid–centroid distances = 3.7579 (15) and 3.7923 (15) Å]. In addition, the bimolecular aggregates which are related by translation along [010] are linked by a further π–π stacking inter­action [centroid–centroid distance = 3.7898 (15) Å], so forming a π-stacked chain running parallel to [010]. However, there are no C—H⋯N hydrogen bonds in the structure nor, despite the number of independent aromatic rings, are there any C—H⋯π hydrogen bonds; hence there are no direction-specific inter­actions between adjacent π-stacked chains.

## Related literature   

For structures of substituted 2-chloro­quinolines, see Insuasty *et al.* (2006 ▸); Hathwar *et al.* (2010 ▸); Anuradha *et al.* (2013*a* ▸,*b* ▸).
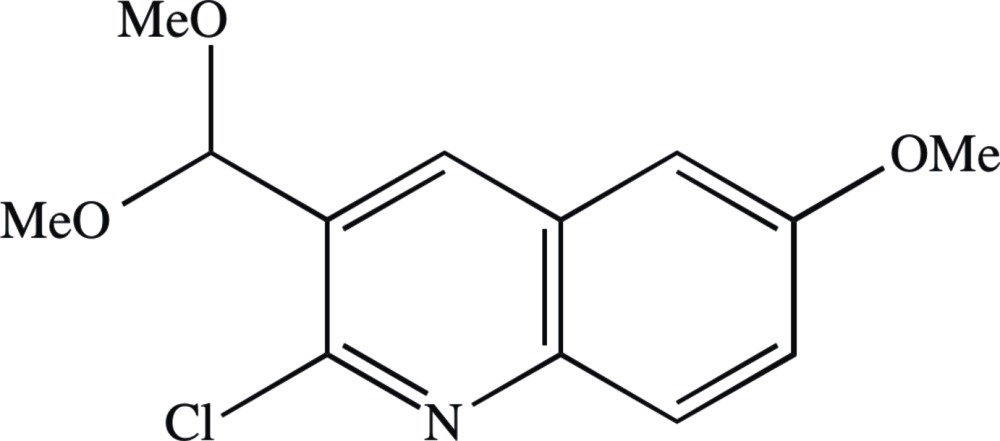


## Experimental   

### Crystal data  

C_13_H_14_ClNO_3_
*M* *_r_* = 267.70
Orthorhombic, 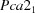

*a* = 27.1156 (9) Å
*b* = 7.1401 (3) Å
*c* = 13.0804 (5) Å
*V* = 2532.47 (17) Å^3^
*Z* = 8
Mo *K*α radiation
μ = 0.30 mm^−1^
*T* = 173 K
0.48 × 0.32 × 0.22 mm

### Data collection  

Agilent Eos Gemini diffractometer
Absorption correction: multi-scan (*CrysAlis RED*; Agilent, 2012 ▸) *T*_min_ = 0.808, *T*_max_ = 0.936
29727 measured reflections
5975 independent reflections
5204 reflections with *I* > 2σ(*I*)
*R*_int_ = 0.037

### Refinement  

*R*[*F* ^2^ > 2σ(*F* ^2^)] = 0.040
*wR*(*F* ^2^) = 0.097
*S* = 1.08
5975 reflections
331 parameters
1 restraint
H-atom parameters constrained
Δρ_max_ = 0.25 e Å^−3^
Δρ_min_ = −0.22 e Å^−3^
Absolute structure: Flack (1983 ▸) *x* determined using 1610 quotients [(*I* ^+^)−(*I* ^−^)]/[(*I* ^+^)+(*I* ^−^)] (Parsons *et al.*, 2013 ▸)
Absolute structure parameter: 0.43 (3)

### 

Data collection: *CrysAlis PRO* (Agilent, 2012 ▸); cell refinement: *CrysAlis PRO*; data reduction: *CrysAlis RED* (Agilent, 2012 ▸); program(s) used to solve structure: *SHELXS97* (Sheldrick, 2008 ▸); program(s) used to refine structure: *SHELXL2014* (Sheldrick, 2015 ▸); molecular graphics: *PLATON* (Spek, 2009 ▸); software used to prepare material for publication: *SHELXL2014* and *PLATON*.

## Supplementary Material

Crystal structure: contains datablock(s) global, I. DOI: 10.1107/S205698901500804X/hg5440sup1.cif

Structure factors: contains datablock(s) I. DOI: 10.1107/S205698901500804X/hg5440Isup2.hkl

Supporting information file. DOI: 10.1107/S205698901500804X/hg5440Isup3.cml

**Fig. 1.**
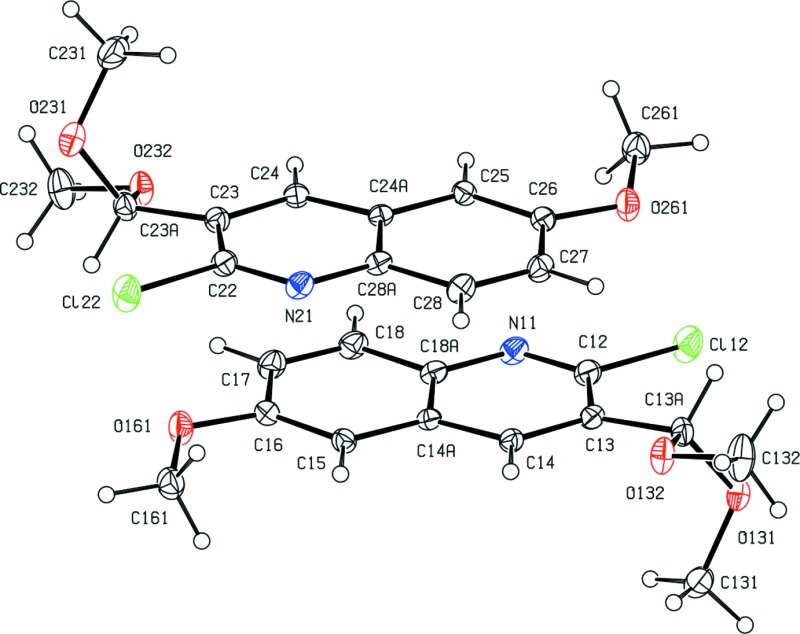
The two independent molecules in the title compound showing the atom-labelling scheme. Displacement ellipsoids are shown at the 30% probability level.

. DOI: 10.1107/S205698901500804X/hg5440fig1.tif
The two independent mol­ecules in the title compound showing the atom-labelling scheme. Displacement ellipsoids are shown at the 30% probability level.

**Fig. 2.**
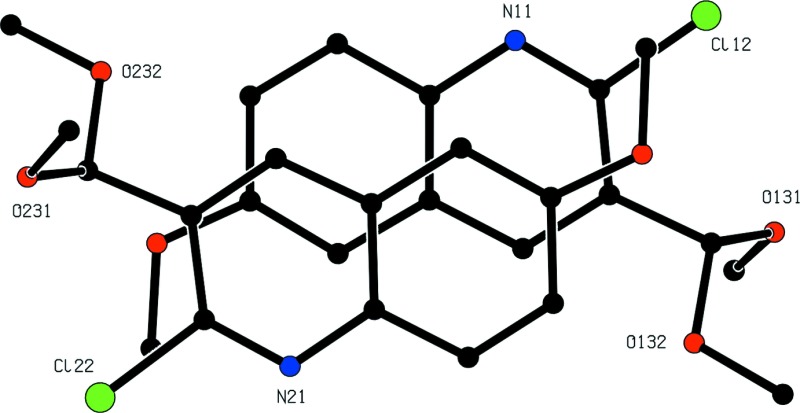
The two molecules in the selected asymmetric unit, viewed normal to the planes of the quinolone units, showing the ring overlap which leads to a π..π sktacking interaction. For the sake of clarity, the H atoms have been omitted.

. DOI: 10.1107/S205698901500804X/hg5440fig2.tif
The two mol­ecules in the selected asymmetric unit, viewed normal to the planes of the quinolone units, showing the ring overlap which leads to a π..π sktacking inter­action. For the sake of clarity, the H atoms have been omitted.

**Fig. 3.**
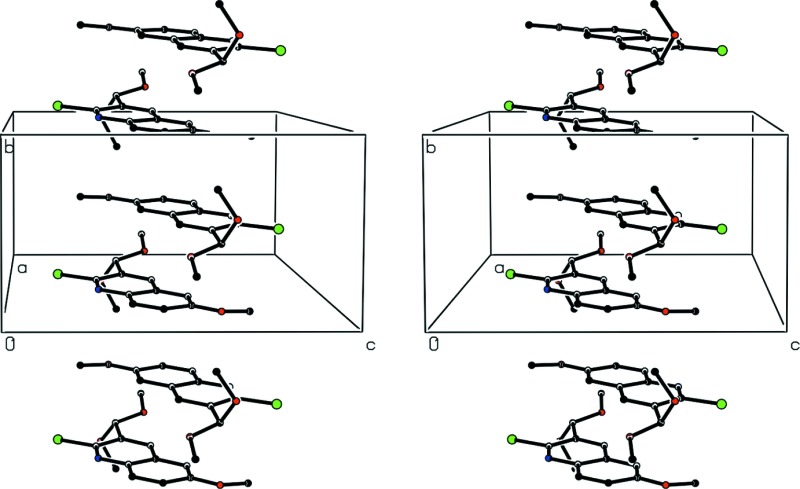
A stereoview of part of the crystal structure of the title compound showing the formation of a π-stacked chain parallel to [010]. For the sake of clarity, the H atoms have been omitted.

. DOI: 10.1107/S205698901500804X/hg5440fig3.tif
A stereoview of part of the crystal structure of the title compound showing the formation of a π-stacked chain parallel to [010]. For the sake of clarity, the H atoms have been omitted.

CCDC reference: 1061227

Additional supporting information:  crystallographic information; 3D view; checkCIF report

## Figures and Tables

**Table 1:** Selected torsion angles ()

C12C13C13*A*O131	69.4(3)
C12C13C13*A*O132	165.7(2)
C13C13*A*O131C131	57.4(3)
C13C13*A*O132C132	170.6(2)
C22C23C23*A*O231	73.3(3)
C22C23C23*A*O232	162.3(2)
C23C23*A*O231C231	58.2(3)
C23C23*A*O232C232	170.3(2)

## supplementary crystallographic information

### S1. Structural commentary

It is convenient to refer to the molecules containing atoms N11 and N21 as molecules of types 1 and 2 respectively. Within the selected asymmetric unit (Fig. 1), the mean planes of the heterocyclic ring of the type 1 molecule and the carbocyclic ring of the type 2 molecule make a dihedral angle of 2.84 (12) °; the ring centroid separation is 3.7579 (15) Å, and the shortest perpendicular distance for the centroid of one ring to the plane of the other is 3.3998 (10) Å, with a ring-centroid offset of *ca* 1.60 Å (Fig. 2). For contact between the carbocylic ring in the type 1 molecule and the heterocyclic ring of the type 2 molecule, the corresponding values are 2.63 (12)°, 3.7923 (15) Å, 3.3993 (11) Å and *ca* 1.68 Å (Fig. 2). In addition, the mean planes of the carbocyclic ring in the type 1 molecule at (*x*, *y*, *z*) and the type 2 molecule at (*x*, -1 + *y*, *z*) make a dihedral angle of only 0.12 (12)°: the ring-centroid separation is 3.7898 (15) Å, the inter­planar spacing is 3.5924 (10) Å, and the ring-centroid offset is *ca* 1.207 Å, leading to the formation of a π-stacked chain of alternating type 1 and type 2 molecules running parallel to the [010] direction (Fig. 3).

### S2. Synthesis and crystallization

Sodium cyano­trohydridoborate (963.9 mg, 15.1 mmol was added in a single portion to a solution of (*E*)-1-((2-chloro-6-meth­oxy­quinolin-3-yl)methyl­ene)-2- (3-fluoro­phenyl)­hydrazine (500 mg, 1.5 mmol) in methanol (20 cm^3^) and the mixture was then stirred for 30 min. The solution was cooled to 273 K and hydrogen chloride solution (16 mol dm^-3^, 4 cm ^3^) was added dropwise during 10 min. Crushed ice was then added followed by the addition of ice-cold water, and the aqueous mixture was exhaustively extracted with ethyl acetate; the combined extracts were dried over anhydrous sodium sulfate, and the organic solvent was removed under educed pressure. The resulting crude product was purified by chromatography on silica gel using a mixture of hexane and ethyl acetate (19:1, *v*/*v*). Crystals of the title compound suitable for single-crystal X-ray diffraction were obtained by slow evaporation, at ambient temperature and in the presence of air, of a solution in hexane-ethyl acetate (1:1, *v*/*v*).

### S3. Refinement

Crystal data, data collection and structure refinement details are summarized in Table 1. All H atoms were located in difference maps and then treated as riding atoms in geometrically idealized positions with C—H distances 0.95 Å (aryl and hetero­aryl) 0.98 Å (methyl) or 1.00 Å (aliphatic CH), and with *U*_iso_(H) = k*U*_eq_(C), where k = 1.5 for the methyl groups, which were permitted to rotate but not to tilt and 1.2 for all other H atoms. The value of the Flack *x* parameter (Flack, 1983) calculated using 1610 quotients of type [(I+)-(I–)]/[(I+)+(I–)] (Parsons *et al.*, 2013), *x* = 0.0.43 (3), indicated partial inversion twinning: the conventional calculation using the TWIN and BASF commands in *SHELXL* gave a less precise value *x* = 0.49 (8).

### Crystal data

**Table:**

C_13_H_14_ClNO_3_	*D*_x_ = 1.404 Mg m^−^^3^
*M**_r_* = 267.70	Mo *K*α radiation, λ = 0.71073 Å
Orthorhombic, *P**c**a*2_1_	Cell parameters from 7046 reflections
*a* = 27.1156 (9) Å	θ = 3.0–32.9°
*b* = 7.1401 (3) Å	µ = 0.30 mm^−^^1^
*c* = 13.0804 (5) Å	*T* = 173 K
*V* = 2532.47 (17) Å^3^	Block, colourless
*Z* = 8	0.48 × 0.32 × 0.22 mm
*F*(000) = 1120	

### Data collection

**Table:**

Agilent Eos Gemini diffractometer	5204 reflections with *I* > 2σ(*I*)
Radiation source: Enhance (Mo) X-ray Source	*R*_int_ = 0.037
ω scans	θ_max_ = 30.0°, θ_min_ = 3.0°
Absorption correction: multi-scan (*CrysAlis RED*; Agilent, 2012)	*h* = −38→38
*T*_min_ = 0.808, *T*_max_ = 0.936	*k* = −10→10
29727 measured reflections	*l* = −18→11
5975 independent reflections	

### Refinement

**Table:**

Refinement on *F*^2^	Hydrogen site location: inferred from neighbouring sites
Least-squares matrix: full	H-atom parameters constrained
*R*[*F*^2^ > 2σ(*F*^2^)] = 0.040	*w* = 1/[σ^2^(*F*_o_^2^) + (0.040*P*)^2^ + 0.6971*P*] where *P* = (*F*_o_^2^ + 2*F*_c_^2^)/3
*wR*(*F*^2^) = 0.097	(Δ/σ)_max_ < 0.001
*S* = 1.08	Δρ_max_ = 0.25 e Å^−^^3^
5975 reflections	Δρ_min_ = −0.22 e Å^−^^3^
331 parameters	Absolute structure: Flack (1983) *x* determined using 1610 quotients [(*I*^+^)-(*I*^-^)]/[(*I*^+^)+(*I*^-^)] (Parsons *et al.*, 2013)
1 restraint	Absolute structure parameter: 0.43 (3)

### Special details

**Table:**

Geometry. All e.s.d.'s (except the e.s.d. in the dihedral angle between two l.s. planes) are estimated using the full covariance matrix. The cell e.s.d.'s are taken into account individually in the estimation of e.s.d.'s in distances, angles and torsion angles; correlations between e.s.d.'s in cell parameters are only used when they are defined by crystal symmetry. An approximate (isotropic) treatment of cell e.s.d.'s is used for estimating e.s.d.'s involving l.s. planes.

### Fractional atomic coordinates and isotropic or equivalent isotropic displacement parameters (Å^2^)

**Table:**

	*x*	*y*	*z*	*U*_iso_*/*U*_eq_	
N11	0.39039 (8)	0.0376 (3)	0.29016 (19)	0.0292 (5)	
C12	0.43758 (10)	0.0736 (4)	0.2853 (2)	0.0288 (6)	
Cl12	0.46105 (3)	0.09675 (13)	0.16196 (6)	0.0454 (2)	
C13	0.46977 (9)	0.0934 (3)	0.3699 (2)	0.0251 (5)	
C14	0.44942 (9)	0.0669 (3)	0.4645 (2)	0.0231 (5)	
H14	0.4696	0.0759	0.5237	0.028*	
C14A	0.39862 (9)	0.0263 (3)	0.4750 (2)	0.0219 (5)	
C15	0.37608 (9)	0.0021 (3)	0.5722 (2)	0.0233 (5)	
H15	0.3953	0.0050	0.6330	0.028*	
C16	0.32597 (9)	−0.0257 (4)	0.5764 (2)	0.0258 (5)	
C17	0.29751 (9)	−0.0318 (4)	0.4865 (3)	0.0319 (6)	
H17	0.2629	−0.0496	0.4913	0.038*	
C18	0.31884 (10)	−0.0127 (4)	0.3927 (3)	0.0305 (6)	
H18	0.2992	−0.0191	0.3326	0.037*	
C18A	0.37043 (9)	0.0167 (4)	0.3853 (2)	0.0249 (5)	
C13A	0.52323 (9)	0.1523 (4)	0.3548 (2)	0.0281 (5)	
H13A	0.5234	0.2665	0.3106	0.034*	
O131	0.55262 (7)	0.0153 (3)	0.30701 (18)	0.0321 (5)	
C131	0.55388 (11)	−0.1605 (4)	0.3590 (3)	0.0429 (8)	
H13B	0.5794	−0.2402	0.3284	0.064*	
H13C	0.5217	−0.2221	0.3527	0.064*	
H13D	0.5614	−0.1400	0.4314	0.064*	
O132	0.54123 (7)	0.2034 (3)	0.45089 (17)	0.0352 (5)	
C132	0.58878 (11)	0.2904 (6)	0.4464 (3)	0.0505 (9)	
H13E	0.5886	0.3884	0.3939	0.076*	
H13F	0.6137	0.1963	0.4292	0.076*	
H13G	0.5966	0.3462	0.5129	0.076*	
O161	0.29952 (6)	−0.0473 (3)	0.66505 (19)	0.0350 (5)	
C161	0.32646 (11)	−0.0603 (4)	0.7579 (2)	0.0359 (6)	
H16A	0.3514	−0.1591	0.7520	0.054*	
H16B	0.3039	−0.0902	0.8140	0.054*	
H16C	0.3427	0.0596	0.7718	0.054*	
N21	0.36627 (8)	0.4764 (3)	0.7052 (2)	0.0283 (5)	
C22	0.31896 (9)	0.4430 (4)	0.7132 (2)	0.0266 (5)	
Cl22	0.29765 (3)	0.42155 (12)	0.83834 (6)	0.04137 (18)	
C23	0.28587 (9)	0.4207 (3)	0.6311 (2)	0.0244 (5)	
C24	0.30472 (9)	0.4451 (4)	0.5349 (2)	0.0232 (5)	
H24	0.2836	0.4347	0.4772	0.028*	
C24A	0.35540 (9)	0.4858 (3)	0.5202 (2)	0.0210 (5)	
C25	0.37671 (9)	0.5086 (3)	0.4222 (2)	0.0230 (5)	
H25	0.3567	0.5039	0.3626	0.028*	
C26	0.42667 (9)	0.5376 (4)	0.4143 (2)	0.0248 (5)	
C27	0.45641 (9)	0.5448 (4)	0.5036 (3)	0.0284 (6)	
H27	0.4910	0.5632	0.4970	0.034*	
C28	0.43643 (9)	0.5259 (4)	0.5976 (2)	0.0293 (6)	
H28	0.4569	0.5329	0.6564	0.035*	
C28A	0.38520 (9)	0.4957 (3)	0.6092 (2)	0.0235 (5)	
C23A	0.23260 (9)	0.3619 (4)	0.6500 (2)	0.0272 (5)	
H23A	0.2329	0.2542	0.6989	0.033*	
O231	0.20304 (7)	0.5042 (3)	0.6925 (2)	0.0374 (5)	
C231	0.20056 (11)	0.6697 (4)	0.6326 (3)	0.0458 (9)	
H23B	0.1889	0.6385	0.5638	0.069*	
H23C	0.2334	0.7264	0.6282	0.069*	
H23D	0.1777	0.7584	0.6645	0.069*	
O232	0.21399 (6)	0.2972 (3)	0.55662 (16)	0.0311 (4)	
C232	0.16713 (11)	0.2090 (5)	0.5660 (3)	0.0424 (8)	
H23E	0.1599	0.1387	0.5034	0.064*	
H23F	0.1417	0.3044	0.5767	0.064*	
H23G	0.1676	0.1231	0.6244	0.064*	
O261	0.45217 (6)	0.5572 (3)	0.32579 (18)	0.0332 (4)	
C261	0.42426 (10)	0.5695 (4)	0.2333 (3)	0.0359 (7)	
H26A	0.4468	0.5846	0.1752	0.054*	
H26B	0.4020	0.6775	0.2368	0.054*	
H26C	0.4049	0.4548	0.2243	0.054*	

### Atomic displacement parameters (Å^2^)

**Table:**

	*U*^11^	*U*^22^	*U*^33^	*U*^12^	*U*^13^	*U*^23^
N11	0.0278 (10)	0.0365 (11)	0.0233 (13)	0.0061 (9)	−0.0032 (9)	−0.0022 (10)
C12	0.0317 (12)	0.0341 (14)	0.0206 (14)	0.0088 (10)	0.0021 (11)	0.0011 (11)
Cl12	0.0410 (4)	0.0734 (5)	0.0217 (3)	0.0135 (4)	0.0051 (3)	0.0014 (4)
C13	0.0240 (11)	0.0266 (12)	0.0248 (13)	0.0051 (9)	0.0023 (10)	0.0012 (10)
C14	0.0211 (11)	0.0261 (12)	0.0221 (13)	0.0026 (9)	−0.0004 (9)	0.0015 (10)
C14A	0.0218 (11)	0.0192 (9)	0.0248 (14)	0.0031 (8)	−0.0001 (10)	0.0008 (10)
C15	0.0225 (10)	0.0247 (12)	0.0226 (14)	−0.0012 (9)	−0.0012 (9)	0.0029 (9)
C16	0.0256 (11)	0.0237 (11)	0.0281 (15)	0.0001 (9)	0.0020 (10)	0.0027 (10)
C17	0.0213 (11)	0.0358 (13)	0.0387 (19)	−0.0008 (10)	−0.0035 (11)	0.0006 (13)
C18	0.0251 (11)	0.0368 (14)	0.0295 (16)	0.0033 (10)	−0.0060 (11)	−0.0002 (12)
C18A	0.0240 (11)	0.0254 (12)	0.0255 (15)	0.0050 (9)	−0.0024 (10)	−0.0008 (10)
C13A	0.0265 (11)	0.0343 (12)	0.0235 (14)	0.0002 (10)	0.0065 (10)	0.0029 (11)
O131	0.0277 (8)	0.0383 (11)	0.0302 (13)	0.0026 (7)	0.0101 (8)	−0.0003 (8)
C131	0.0310 (14)	0.0372 (15)	0.061 (2)	0.0050 (12)	0.0101 (14)	0.0021 (15)
O132	0.0266 (9)	0.0515 (12)	0.0276 (11)	−0.0055 (8)	0.0059 (8)	−0.0048 (9)
C132	0.0339 (15)	0.072 (2)	0.046 (2)	−0.0184 (15)	0.0083 (14)	−0.0148 (18)
O161	0.0236 (8)	0.0496 (11)	0.0318 (13)	−0.0054 (8)	0.0028 (8)	0.0062 (11)
C161	0.0329 (14)	0.0461 (16)	0.0286 (16)	−0.0009 (12)	0.0025 (12)	0.0056 (13)
N21	0.0260 (10)	0.0350 (12)	0.0237 (12)	0.0051 (9)	−0.0022 (9)	−0.0026 (10)
C22	0.0287 (12)	0.0329 (13)	0.0181 (13)	0.0054 (10)	0.0008 (10)	0.0005 (10)
Cl22	0.0379 (3)	0.0660 (5)	0.0201 (3)	0.0034 (3)	0.0030 (3)	0.0008 (4)
C23	0.0248 (11)	0.0255 (11)	0.0227 (13)	0.0003 (9)	0.0008 (10)	−0.0002 (9)
C24	0.0216 (10)	0.0261 (11)	0.0219 (13)	0.0007 (9)	−0.0029 (10)	0.0014 (10)
C24A	0.0224 (10)	0.0175 (10)	0.0231 (13)	0.0016 (8)	−0.0003 (10)	−0.0010 (9)
C25	0.0227 (11)	0.0233 (12)	0.0230 (14)	−0.0014 (8)	−0.0013 (10)	0.0017 (9)
C26	0.0236 (11)	0.0232 (11)	0.0277 (15)	−0.0008 (9)	0.0007 (10)	0.0017 (11)
C27	0.0204 (10)	0.0311 (12)	0.0337 (16)	−0.0019 (9)	−0.0024 (10)	−0.0015 (12)
C28	0.0220 (11)	0.0349 (13)	0.0310 (16)	−0.0011 (10)	−0.0059 (11)	−0.0043 (12)
C28A	0.0238 (11)	0.0237 (11)	0.0231 (14)	0.0037 (9)	−0.0029 (10)	−0.0030 (10)
C23A	0.0261 (11)	0.0340 (12)	0.0214 (13)	−0.0018 (9)	0.0021 (10)	0.0036 (11)
O231	0.0294 (9)	0.0439 (12)	0.0387 (14)	0.0014 (8)	0.0089 (9)	−0.0039 (10)
C231	0.0265 (13)	0.0388 (15)	0.072 (3)	0.0013 (11)	0.0056 (15)	0.0013 (16)
O232	0.0241 (8)	0.0432 (11)	0.0260 (11)	−0.0084 (8)	0.0022 (7)	0.0010 (8)
C232	0.0353 (15)	0.0542 (18)	0.0378 (18)	−0.0194 (13)	0.0039 (13)	−0.0012 (14)
O261	0.0236 (8)	0.0471 (11)	0.0288 (12)	−0.0042 (8)	0.0034 (8)	0.0046 (10)
C261	0.0320 (14)	0.0484 (17)	0.0275 (16)	0.0008 (12)	0.0011 (11)	0.0028 (14)

### Geometric parameters (Å, º)

**Table:**

N11—C12	1.307 (3)	N21—C22	1.309 (3)
N11—C18A	1.365 (4)	N21—C28A	1.363 (4)
C12—C13	1.417 (4)	C22—C23	1.408 (4)
C12—Cl12	1.742 (3)	C22—Cl22	1.743 (3)
C13—C14	1.368 (4)	C23—C24	1.370 (4)
C13—C13A	1.522 (3)	C23—C23A	1.524 (3)
C14—C14A	1.414 (3)	C24—C24A	1.417 (3)
C14—H14	0.9500	C24—H24	0.9500
C14A—C18A	1.402 (4)	C24A—C25	1.416 (4)
C14A—C15	1.422 (4)	C24A—C28A	1.419 (4)
C15—C16	1.374 (3)	C25—C26	1.374 (3)
C15—H15	0.9500	C25—H25	0.9500
C16—O161	1.372 (4)	C26—O261	1.355 (3)
C16—C17	1.407 (4)	C26—C27	1.420 (4)
C17—C18	1.364 (5)	C27—C28	1.351 (4)
C17—H17	0.9500	C27—H27	0.9500
C18—C18A	1.418 (3)	C28—C28A	1.414 (3)
C18—H18	0.9500	C28—H28	0.9500
C13A—O132	1.396 (3)	C23A—O232	1.400 (3)
C13A—O131	1.408 (3)	C23A—O231	1.408 (3)
C13A—H13A	1.0000	C23A—H23A	1.0000
O131—C131	1.428 (4)	O231—C231	1.419 (4)
C131—H13B	0.9800	C231—H23B	0.9800
C131—H13C	0.9800	C231—H23C	0.9800
C131—H13D	0.9800	C231—H23D	0.9800
O132—C132	1.433 (3)	O232—C232	1.424 (3)
C132—H13E	0.9800	C232—H23E	0.9800
C132—H13F	0.9800	C232—H23F	0.9800
C132—H13G	0.9800	C232—H23G	0.9800
O161—C161	1.420 (4)	O261—C261	1.430 (4)
C161—H16A	0.9800	C261—H26A	0.9800
C161—H16B	0.9800	C261—H26B	0.9800
C161—H16C	0.9800	C261—H26C	0.9800
			
C12—N11—C18A	117.0 (2)	C22—N21—C28A	117.4 (2)
N11—C12—C13	125.8 (3)	N21—C22—C23	125.7 (3)
N11—C12—Cl12	114.9 (2)	N21—C22—Cl22	114.6 (2)
C13—C12—Cl12	119.3 (2)	C23—C22—Cl22	119.7 (2)
C14—C13—C12	116.4 (2)	C24—C23—C22	116.6 (2)
C14—C13—C13A	122.7 (2)	C24—C23—C23A	122.5 (2)
C12—C13—C13A	120.9 (3)	C22—C23—C23A	120.7 (2)
C13—C14—C14A	120.6 (2)	C23—C24—C24A	120.8 (2)
C13—C14—H14	119.7	C23—C24—H24	119.6
C14A—C14—H14	119.7	C24A—C24—H24	119.6
C18A—C14A—C14	117.4 (3)	C25—C24A—C24	122.8 (2)
C18A—C14A—C15	120.5 (2)	C25—C24A—C28A	120.3 (2)
C14—C14A—C15	122.0 (2)	C24—C24A—C28A	116.8 (3)
C16—C15—C14A	118.6 (3)	C26—C25—C24A	119.2 (3)
C16—C15—H15	120.7	C26—C25—H25	120.4
C14A—C15—H15	120.7	C24A—C25—H25	120.4
O161—C16—C15	124.5 (3)	O261—C26—C25	125.6 (3)
O161—C16—C17	114.6 (2)	O261—C26—C27	114.1 (2)
C15—C16—C17	120.9 (3)	C25—C26—C27	120.2 (3)
C18—C17—C16	121.1 (2)	C28—C27—C26	121.2 (2)
C18—C17—H17	119.5	C28—C27—H27	119.4
C16—C17—H17	119.5	C26—C27—H27	119.4
C17—C18—C18A	119.7 (3)	C27—C28—C28A	120.4 (3)
C17—C18—H18	120.2	C27—C28—H28	119.8
C18A—C18—H18	120.2	C28A—C28—H28	119.8
N11—C18A—C14A	122.8 (2)	N21—C28A—C28	118.9 (2)
N11—C18A—C18	118.0 (3)	N21—C28A—C24A	122.4 (2)
C14A—C18A—C18	119.2 (3)	C28—C28A—C24A	118.6 (3)
O132—C13A—O131	112.5 (2)	O232—C23A—O231	112.2 (2)
O132—C13A—C13	106.8 (2)	O232—C23A—C23	106.9 (2)
O131—C13A—C13	113.9 (2)	O231—C23A—C23	113.9 (2)
O132—C13A—H13A	107.8	O232—C23A—H23A	107.9
O131—C13A—H13A	107.8	O231—C23A—H23A	107.9
C13—C13A—H13A	107.8	C23—C23A—H23A	107.9
C13A—O131—C131	114.4 (2)	C23A—O231—C231	114.2 (3)
O131—C131—H13B	109.5	O231—C231—H23B	109.5
O131—C131—H13C	109.5	O231—C231—H23C	109.5
H13B—C131—H13C	109.5	H23B—C231—H23C	109.5
O131—C131—H13D	109.5	O231—C231—H23D	109.5
H13B—C131—H13D	109.5	H23B—C231—H23D	109.5
H13C—C131—H13D	109.5	H23C—C231—H23D	109.5
C13A—O132—C132	113.0 (2)	C23A—O232—C232	113.1 (2)
O132—C132—H13E	109.5	O232—C232—H23E	109.5
O132—C132—H13F	109.5	O232—C232—H23F	109.5
H13E—C132—H13F	109.5	H23E—C232—H23F	109.5
O132—C132—H13G	109.5	O232—C232—H23G	109.5
H13E—C132—H13G	109.5	H23E—C232—H23G	109.5
H13F—C132—H13G	109.5	H23F—C232—H23G	109.5
C16—O161—C161	117.47 (19)	C26—O261—C261	117.32 (19)
O161—C161—H16A	109.5	O261—C261—H26A	109.5
O161—C161—H16B	109.5	O261—C261—H26B	109.5
H16A—C161—H16B	109.5	H26A—C261—H26B	109.5
O161—C161—H16C	109.5	O261—C261—H26C	109.5
H16A—C161—H16C	109.5	H26A—C261—H26C	109.5
H16B—C161—H16C	109.5	H26B—C261—H26C	109.5
			
C18A—N11—C12—C13	−0.3 (4)	C28A—N21—C22—C23	1.6 (4)
C18A—N11—C12—Cl12	179.47 (19)	C28A—N21—C22—Cl22	−179.65 (19)
N11—C12—C13—C14	2.0 (4)	N21—C22—C23—C24	−3.3 (4)
Cl12—C12—C13—C14	−177.72 (19)	Cl22—C22—C23—C24	178.01 (19)
N11—C12—C13—C13A	−174.6 (3)	N21—C22—C23—C23A	173.5 (3)
Cl12—C12—C13—C13A	5.7 (3)	Cl22—C22—C23—C23A	−5.2 (3)
C12—C13—C14—C14A	−1.4 (3)	C22—C23—C24—C24A	1.6 (4)
C13A—C13—C14—C14A	175.1 (2)	C23A—C23—C24—C24A	−175.1 (2)
C13—C14—C14A—C18A	−0.7 (3)	C23—C24—C24A—C25	178.7 (2)
C13—C14—C14A—C15	−178.6 (2)	C23—C24—C24A—C28A	1.3 (4)
C18A—C14A—C15—C16	−1.8 (4)	C24—C24A—C25—C26	−176.4 (2)
C14—C14A—C15—C16	176.1 (2)	C28A—C24A—C25—C26	1.0 (3)
C14A—C15—C16—O161	−178.7 (2)	C24A—C25—C26—O261	178.4 (2)
C14A—C15—C16—C17	0.6 (4)	C24A—C25—C26—C27	−0.1 (4)
O161—C16—C17—C18	−179.9 (3)	O261—C26—C27—C28	−179.5 (3)
C15—C16—C17—C18	0.8 (4)	C25—C26—C27—C28	−0.8 (4)
C16—C17—C18—C18A	−1.0 (4)	C26—C27—C28—C28A	0.9 (4)
C12—N11—C18A—C14A	−2.1 (4)	C22—N21—C28A—C28	−178.0 (2)
C12—N11—C18A—C18	176.8 (2)	C22—N21—C28A—C24A	1.7 (4)
C14—C14A—C18A—N11	2.6 (4)	C27—C28—C28A—N21	179.7 (3)
C15—C14A—C18A—N11	−179.5 (2)	C27—C28—C28A—C24A	0.0 (4)
C14—C14A—C18A—C18	−176.3 (2)	C25—C24A—C28A—N21	179.4 (2)
C15—C14A—C18A—C18	1.6 (4)	C24—C24A—C28A—N21	−3.1 (3)
C17—C18—C18A—N11	−179.2 (3)	C25—C24A—C28A—C28	−0.9 (3)
C17—C18—C18A—C14A	−0.2 (4)	C24—C24A—C28A—C28	176.6 (2)
C14—C13—C13A—O132	−10.6 (3)	C24—C23—C23A—O232	14.3 (3)
C12—C13—C13A—O131	−69.4 (3)	C22—C23—C23A—O231	73.3 (3)
C12—C13—C13A—O132	165.7 (2)	C22—C23—C23A—O232	−162.3 (2)
C14—C13—C13A—O131	114.2 (3)	C24—C23—C23A—O231	−110.1 (3)
O132—C13A—O131—C131	64.3 (3)	O232—C23A—O231—C231	−63.4 (3)
C13—C13A—O131—C131	−57.4 (3)	C23—C23A—O231—C231	58.2 (3)
O131—C13A—O132—C132	63.7 (3)	O231—C23A—O232—C232	−64.2 (3)
C13—C13A—O132—C132	−170.6 (2)	C23—C23A—O232—C232	170.3 (2)
C15—C16—O161—C161	−6.5 (4)	C25—C26—O261—C261	7.3 (4)
C17—C16—O161—C161	174.2 (3)	C27—C26—O261—C261	−174.2 (2)
