# Supplementary figures and images for: Crystal structure of 2-chloro-3-(di­meth­oxy­meth­yl)-6-meth­oxy­quinoline
Source: Acta Crystallogr E Crystallogr Commun. 2015 Apr 30;71(Pt 5):o364–5. doi: 10.1107/S205698901500804X (PMC4420130; doi:10.1107/S205698901500804X)

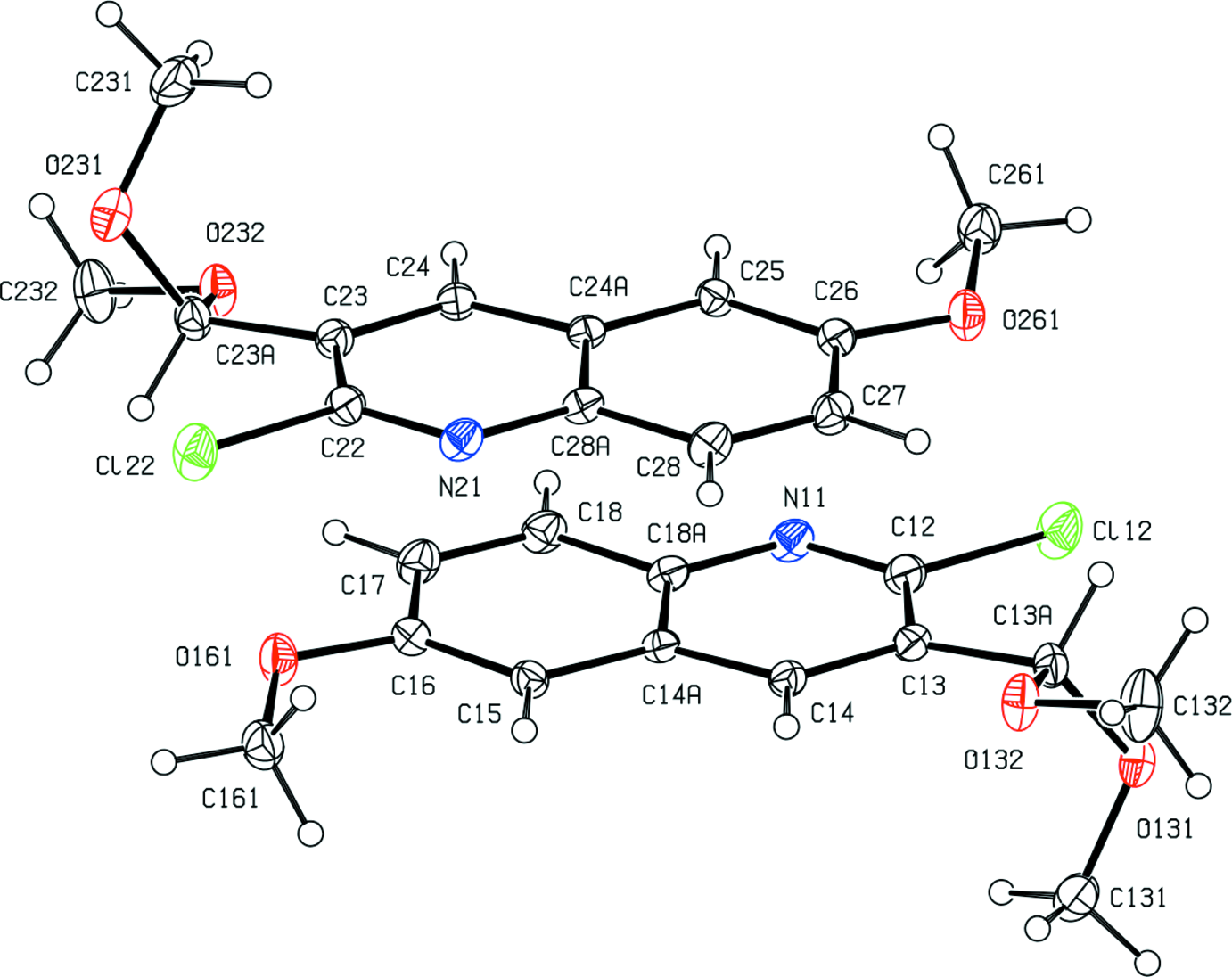

Supplement: Supplementary file 4 [file e-71-0o364-fig1.tif]

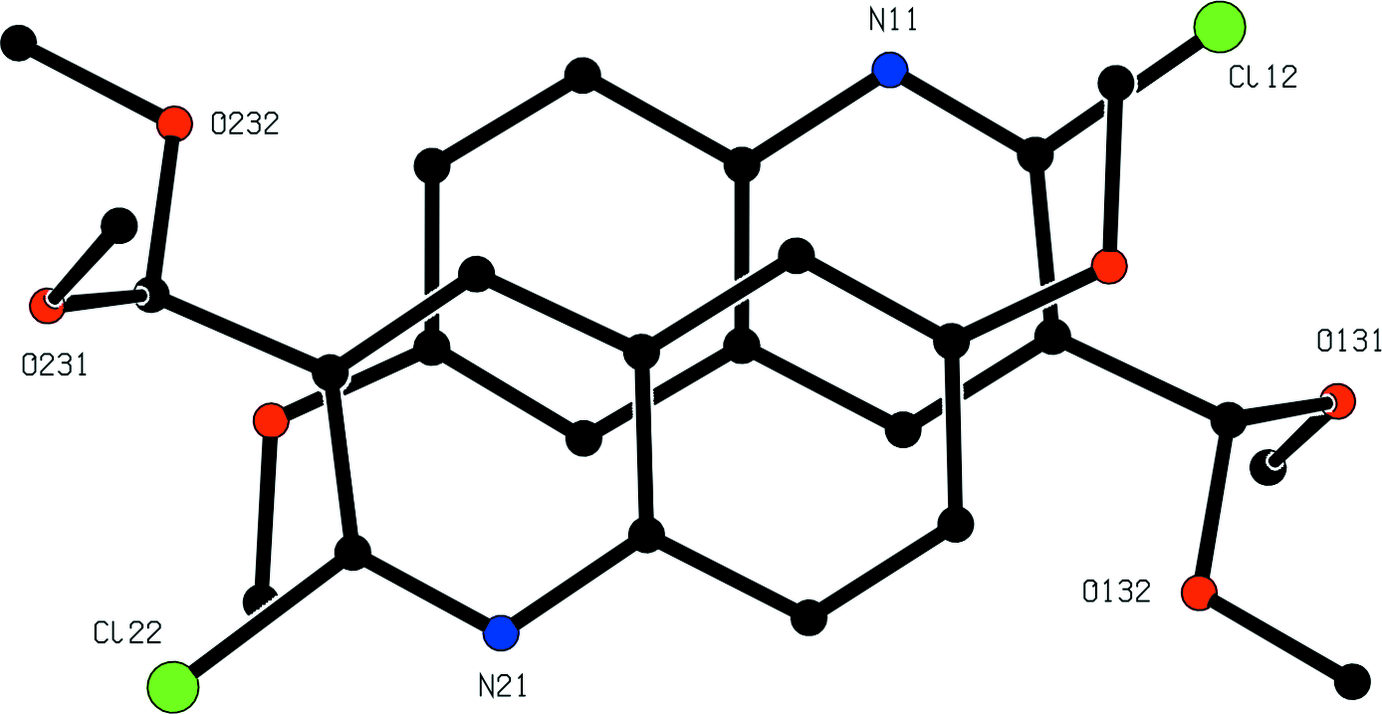

Supplement: Supplementary file 5 [file e-71-0o364-fig2.tif]

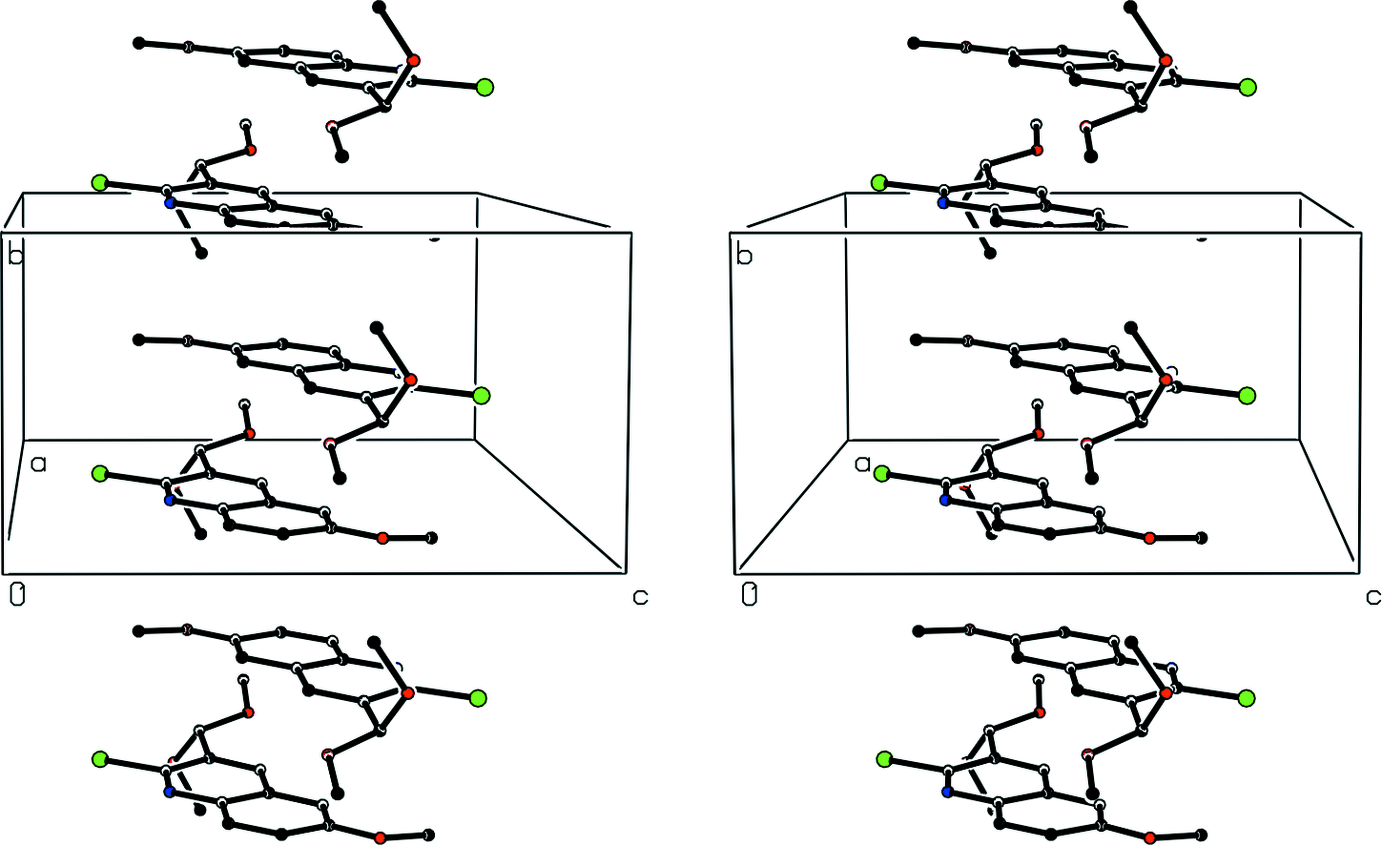

Supplement: Supplementary file 6 [file e-71-0o364-fig3.tif]
